# A clinical field trial on wound healing after disbudding of dairy calves with or without antimicrobial spray

**DOI:** 10.3389/fvets.2025.1445605

**Published:** 2025-04-07

**Authors:** H. J. C. Bijkerk, M. Nielen, I. M. van Geijlswijk, J. C. M. Vernooij, R. Jorritsma

**Affiliations:** ^1^Sustainable Ruminant Health Group, Faculty of Veterinary Medicine, Utrecht University, Utrecht, Netherlands; ^2^Farm Animal Health Division, Faculty of Veterinary Medicine, Utrecht, Netherlands; ^3^Department of Pharmacy, Faculty of Veterinary Medicine, Institute of Risk Assessment Sciences, Utrecht University, Utrecht, Netherlands

**Keywords:** wound healing, topical (local) antibiotics, non-inferiority, calves, Keno™Fix, disbudding

## Abstract

Calves are routinely disbudded with hot iron disbudders in Netherlands. Disbudding wounds are frequently treated with antimicrobial wound spray with the aim of preventing infection and improving wound healing. Given the policy for the prudent use of antimicrobials, we decided to evaluate the effect of antimicrobial wound spray vs. non-antimicrobial wound spray after disbudding in a commercial setting. In this field trial, we treated 255 calves after disbudding with chlortetracycline (CTC) spray and Keno™Fix (KF) on alternating the left and right horn buds. At 1 week and 4 weeks after disbudding, we measured the healing rate (HR) in mm/week and the lesion score (LS) of the wounds. The field conditions on farms were also monitored and investigated for an effect on HR and LS. The HR of KF wounds was numerically lower than CTC wounds but did not pass the non-inferiority threshold of 0.5 mm/week. Lesion scores were worse for KF-treated wounds than for CTC-treated wounds. For both CTC and KF wounds, we found that a worse lesion score was associated with a lower HR. We found that lesion scores were worse for KF-treated wounds and calculated that five calves need to be treated with CTC to prevent one calf from developing an inferior wound score. Since the HR of KF was lower, though not inferior to CTC-treated wounds, we conclude that in certain circumstances, the use of an antibiotic spray after cautery disbudding of calves may not be necessary.

## Introduction

Disbudding of calves is a common practice in the dairy industry to prevent injuries among the animals and the animal caretakers on the farm. Calves can be disbudded chemically with caustic paste, a scoop disbudder, or a hot iron. Dutch law requires the use of anesthesia during disbudding and only allows hot-iron disbudding. These devices operate either on butane gas or electricity. By European law (1, 2), anesthesia is required. It is standard practice in Netherlands to sedate the calves with an alpha-2-agonist, apply a local anesthetic block around the cornual nerve, and use systemic analgesia with an NSAID after disbudding. No specific treatment for the resulting wound is mandated by the EU or Dutch law.

Wound healing is a complex process encompassing multiple phases, each with underlying biochemical mechanisms. A single wound can display multiple phases simultaneously over the wound surface, depending on wound size, local infection, and other complications (3–6).

Disbudded horns are often treated with wound sprays to aid healing and prevent and/or treat infection. Some sprays that are often used contain chlortetracycline (CTC) and oxytetracycline (OTC), while there are also sprays and treatments without antimicrobials such as Aluspray (ALU), Keno™Fix, Acederm, and topical anesthetic (TA) gels. Stilwell and Laven (7) investigated the effect of OTC vs. TA. They concluded that TA might speed up wound healing compared to OTC. Ridgway et al. (8) found that antimicrobial treatment offers an advantage over no treatment during the first 14 days, but no significant difference was observed after 14 days. This suggests that antimicrobial treatment is effective in the early stages of wound healing. Huebner et al. (9) used a wound scoring system to evaluate wound healing of ALU-treated wounds compared to no treatment. They found that using ALU improved healing at 3 weeks after disbudding and that wounds with a poor wound score were at a higher risk of a poor wound score on subsequent visits. To the best of our knowledge, the effect of wound score on the speed of wound healing is not known.

Keno™Fix is one of the antiseptic wound sprays authorized for claw disinfection, often used to disinfect disbudding wounds; it contains the disinfectant chlorocresol 1.5% w/w and denatonium (bitter taste, to avert licking), and the manufacturer claims that the spray creates a protective oxygen-permeable film on the skin, releasing chlorocresol during a period of time. The product is dyed bright blue, reminiscent of blue antimicrobial sprays. To the best of our knowledge, no studies on the effect of Keno™Fix on disbudding wound healing have been performed as of the time of writing.

We therefore decided to conduct a clinical non-inferiority trial to evaluate whether Keno™Fix provides a suitable alternative to antimicrobial spray. To this end, we applied CTC spray on one horn of 255 calves after disbudding and Keno™Fix on the other horn. We followed these calves for 4 weeks after disbudding, recording wound size using calipers and lesion scores using a scoring method developed by Huebner et al. (9). We used the wound size over time to calculate the wound healing rate as our primary outcome parameter.

## Materials and methods

### Participants

Two veterinary practices assisted in the enrollment of farmers from 7 October 2019 to 17 January 2020. The practices agreed to ask all farmers with scheduled farm visits to participate in this study. When the farmers agreed to do so, all heifer calves that were disbudded during this farm visit were included in the study. Only calves before 8 weeks of age were disbudded, in accordance with Dutch law (10). As the expected effect size of both treatments was not available, we arbitrarily aimed to include at least 200 calves.

### Interventions

All calves were disbudded under the supervision of the local veterinarian. The calves were sedated using an alpha-2-agonist, and a local anesthetic block was placed at the corneal nerve. The horn bud was removed in all calves. They used one of the following types of disbudders: (I) the butane-fueled Portasol III (Oglesby & Butler Ltd., Carlow, Ireland) with a diameter of 17 mm or (II) an electric disbudder (Lister Disbudder HLBA 230V GmbH, Lüdenscheid, Germany) with a diameter of 18 mm. Each calf was treated with Keno™Fix (KF, Cid Lines N.V., Ieper, Belgium) on one horn bud and CTC-Spray 2.45%w/w (Dechra B.V., Bladel, The Netherlands) on the other horn bud. Treatment was allocated using a coin flip to determine whether the first calf of a pen would receive CTC on the left or right horn; for the subsequent calves in the pen, the treatment was alternated. Both products were sprayed for 3 s and at a distance of 15–20 cm, according to the manufacturer’s instructions.

### Blinding

The disbudding operators were blinded for the treatment when they disbudded the calves as treatments were applied after disbudding. We assume that scoring of the lesions in subsequent weeks should be regarded as blind as the calves were often moved between pens; both treatments give a similar blue color to the lesion, and previous recordings were not available at the time of scoring.

### Measurements

The farms were visited three times in a 4-week period to collect data: at disbudding, 1 week after disbudding, and 4 weeks after disbudding. The 4-week period was chosen for practical considerations so the effect of a single spray is expected to be diluted if a longer recording interval is chosen. Calf number, farm number, disbudding operator, disbudding iron, treatment, and observer were recorded at the first visit. During the visits in weeks 1 and 4, the lesion score and diameter of the wounds were recorded.

During all three visits, participant background data were collected. The background data recorded were the hygiene score of the pen, the risk of bumping the head into the feeding fence, and the housing type. Hygiene was scored on a scale of 1–4 at each visit. Score 1 indicated a clean and dry pen, with no marks of manure and fresh, dry bedding. Score 2 indicated a clean pen, without manure on the walls or visible on the bedding, but with wet bedding. Score 3 indicated limited soiling and a wet pen, with some manure on the wall or bedding. Score 4 indicated that a lot of manure was present on bedding and/or walls. Pens with animals with diarrhea also received a score of 4. Calf pens were scored on the risk of a calf bumping its head against the fence during eating or drinking as present or absent (Y/N). The housing type was recorded as solitary or group housing.

The lesion score (LS) was registered according to Huebner et al. (9), with LS = 1 for a wound without scab or discharge, LS = 2 for a crusted-filled wound with a scab or raised scabs present, and LS = 3 for a wound with dried or moist purulent exudate. As the number of observations with a lesion score of 3 was limited, this category collapsed with a lesion score of 2 in the statistical analyses. The diameter of the wound was measured using calipers at the widest diameter of the wound, according to Huebner et al. (9). To prevent wound contamination, we did not measure the diameter of the wound directly after disbudding (0 weeks) but assumed that the diameter at disbudding was equal to the diameter of the disbudding iron.

One of the two student researchers performed all observations. These persons regularly exchanged photos of the scores of horn buds and wounds to facilitate uniform scoring between observers. Each farm was visited by the same researcher over the course of the enrolled period. These students also performed the disbudding on some farms.

### Statistical methods

The primary outcome parameter was the change in wound diameter per week and was calculated as the healing rate in mm/week for each horn bud (change in wound diameter between week 0 and week 4 divided by 4). The healing rate was used in the assessment of non-inferiority. A difference in healing rate between KF and CTC of 0.5 mm/week was determined as the non-inferiority margin. The outcome healing rate was analyzed using a linear mixed-effects model (library lme4) with a random intercept for the farm and calf within the farm to account for the correlation between the observations. The explanatory variables in the full model were treatment; disbudder type; disbudding operator; the hygiene score at disbudding, at 1 week, and at 4 weeks after disbudding; head bump risk; and housing type. A stepwise backward procedure with maximum likelihood estimation based on the Akaike Information Criterion (AIC) was used to select the best-fitting but most parsimonious model. The final model was estimated by the REML. A variable was removed when the AIC was lower in the smaller model or at most 2 points higher than in the extended model. In the final model, only treatment and disbudding operators were present. The validity of the model was studied using the residuals of the full model (library DHARMa) for normality and homoscedasticity. No abnormalities were found.

The secondary outcome parameter lesion score of the wound was analyzed as the presence or absence of an LS > 1 wound using a mixed-effects logistic regression model (library lme4) with a random intercept for the farm and calf within the farm to account for the correlation between the observations. Two models were created and analyzed, one for the wounds at 1 week after disbudding and 1 for the wounds at 4 weeks after disbudding. Explanatory variables in the full model were the same as in the previously described model. The model’s validity was studied using the library DHARMa, showing a reasonably fitting model with the data for the model at week 1. The model at week 4 did not converge and was not analyzed. The same stepwise backward selection procedure was applied as previously described. In the final model, the treatment and disbudding operator remained in the model to answer the research question about changes in the risk of infected wounds over time. The model results were presented as adjusted odds ratios with a 95% confidence interval, along with crude, unadjusted odds ratios and a 95% confidence interval. The risk difference was calculated based on the crude risks with a 95% confidence interval, while the predicted risk was derived using the model without a confidence interval. A risk difference of 10% or higher was considered clinically relevant.

R Studio (2021.09.0 Build 351) and R (version 4.1.2) were used for all statistical analyses.

## Results

A total of 37 farms were visited for disbudding. In total, 255 calves were enrolled in the study. Two farms were included in the study twice, with two groups of calves each. Farms had between 1 and 35 (median = 4) calves available for disbudding. Calves were between 2 weeks and 2 months old at the time of disbudding. In 132 calves of the 255 calves, the right wound was treated with KF, and in the remaining calves, the left wound was treated with KF. The other wound was treated with CTC. Table 1 provides the baseline results of wound healing parameters per horn.

**Table 1:** Baseline results of wound size, lesion score, and healing parameters per horn.

	CTC^a^	KF^a^	Difference (CTC – KF)
Lesion score^b^ 1 week post disbudding			
LS = 1, *n* (%)	229 (90%)	178 (70%)	51
LS = 2, *n* (%)	26 (10%)	60 (24%)	−34
LS = 3, *n* (%)	0	17 (7%)	−17
Lesion score 4 weeks post disbudding			
LS = 1, *n* (%)	224 (88%)	197 (77%)	27
LS = 2, *n* (%)	28 (11%)	46 (18%)	−18
LS = 3, *n* (%)	3 (1%)	12 (5%)	−9
Mean wound diameter			
Week 1, mm (CI)	14.8 (14.5; 15.0)	14.9 (14.6; 15.1)	−0.1 (−0.4; 0.1)
Week 4, mm (CI)	11.0 (10.6; 11.5)	11.5 (11.0; 12.1)	−0.5 (−1.0; 0.0)
Mean wound healing rate			
Week 0–1, mm/week (CI)	3.0 (2.7; 3.2)	2.8 (2.6; 3.1)	0.1 (−0.1; 0.4)
Week 1–4, mm/week (CI)	1.2 (1.1; 1.4)	1.1 (0.9; 1.3)	0.1 (0; 0.3)
Week 0–4, mm/week (CI)	1.7 (1.6; 1.8)	1.5 (1.4; 1.7)	0.1 (0; 0.3)

a CTC, chlortetracycline spray; KF, Keno™Fix spray.

b Lesion score was recorded according to Huebner et al. (9). LS, lesion score; CI, confidence interval; *n*, number.

### Non-inferiority of KF compared to CTC for wound healing

**Figure 1:**
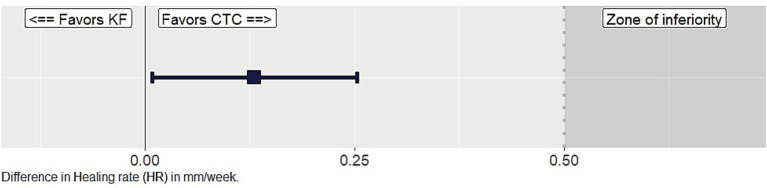
Mean difference in healing rate (in mm/week) between chlortetracycline (CTC)-treated wounds and Keno™Fix-treated wounds (KF) based on the adjusted model, with the farm as a random variable. The box indicates the mean, with a 95% confidence interval displayed in the bracket. The grayed-out area indicates the zone of inferiority of Keno™Fix at 0.5 mm/week.

The adjusted mean difference in HR and the 95% confidence interval between CTC-treated and KF-treated wounds over the study period is displayed in Figure 1. The confidence interval of the difference in healing rate between weeks 0 and 4 is larger than 0, indicating a significantly lower healing rate for KF-treated wounds. However, the lower HR for KF-treated wounds did not pass the margin of inferiority.

KF-treated wounds had a significantly higher prevalence of LS > 1 at both 1 week and 4 weeks after disbudding (Table 2). At both time points, the difference in the risk of a higher lesion score exceeded the margin of clinical relevance of 10%. Based on the prevalence difference between the CTC and KF groups, 5 calves would need to be treated with CTC to prevent 1 calf from developing LS > 1 at 1 week after disbudding, and 10 calves to prevent 1 calf from developing LS > 1 at 4 weeks after disbudding.

**Table 2:** The odds ratios and risks for a lesion score > 1 per treatment.

	Crude odds ratio (CI)	Crude risk (CI)	Adjusted odds ratio (CI)	Adjusted risk
KF^a^ week 1	3.81 (2.34; 6.19)	30.2% (24.7; 36.3)	4.47 (2.59; 7.74)	29.9%
CTC^a^ week 1		10.2% (6.9; 14.7)		8.8%
Risk difference		20.0% (13.3; 26.7)		20.4%
KF week 4	2.13 (1.32; 3.42)	22.3 (17.8; 28.5)	NA^b^	
CTC week 4		12.2 (8.5; 17.0)		
Risk difference		10.1 (3.9; 17.2)		

Odds ratios and risk difference for a lesion score > 1. The lesion score was recorded according to Huebner et al. (9). Odds ratios are displayed as the odds of KF compared to CTC. The risk difference was calculated as the risk of Ketofen–the risk of CTC. Crude odds ratios and risks were calculated without adjustment. Adjusted odds ratio and risks were calculated using a logistic regression model with farm and animal numbers nested within the farm as random effects.

a KF, Keno™Fix spray; CTC, chlortetracycline spray.

b The model for lesion score per treatment at week 4 did not converge, so no conclusions can be drawn on the adjusted model.

**Figure 2:**
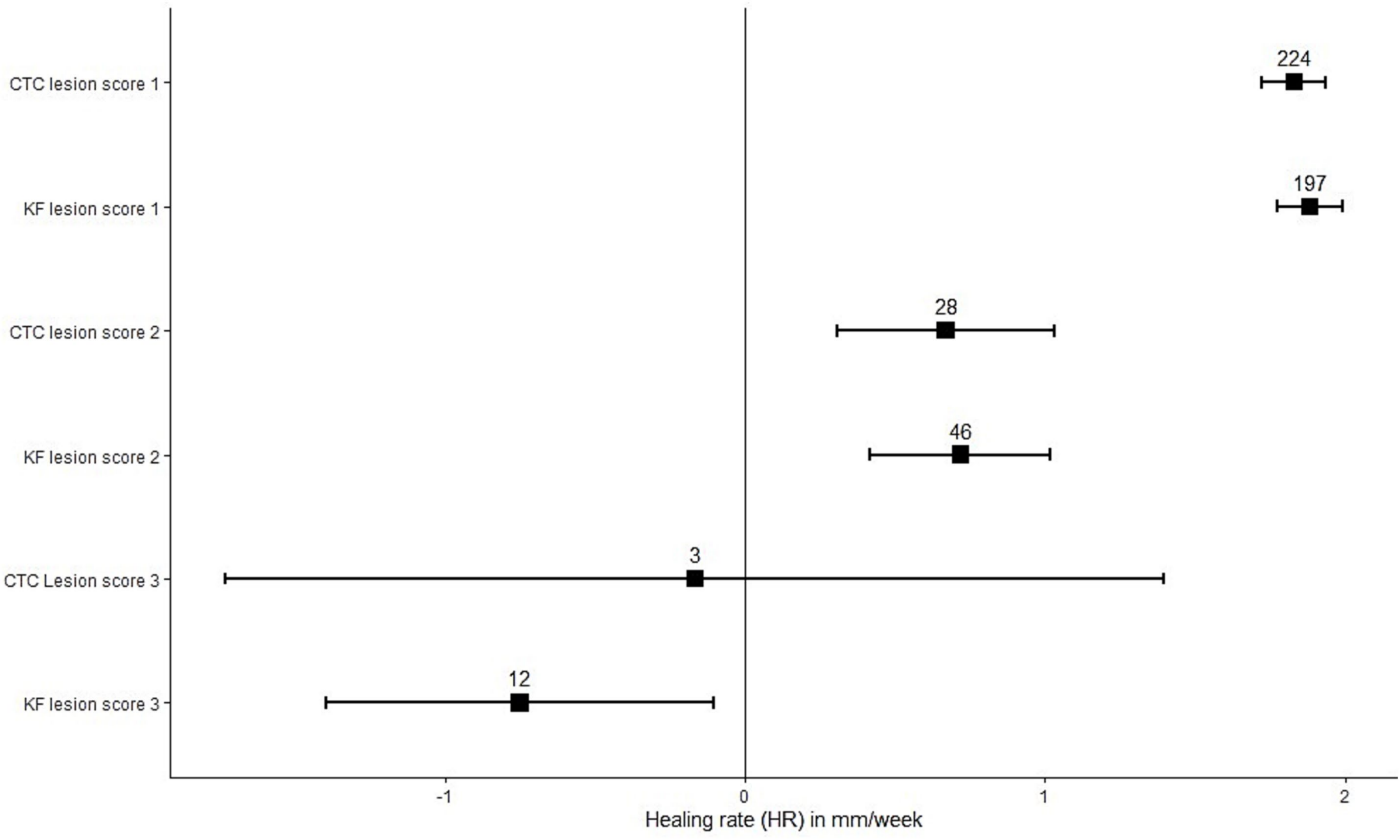
Mean healing rate in mm/week for each lesion score at week 4, per treatment group. KF indicates Keno™Fix-treated wounds, and CTC indicates chlortetracycline-treated wounds. The boxes indicate the means, with the confidence interval displayed by the error bars. The numbers indicate the number of wounds in each group.

Figure 2 displays the correlation between LS and HR. We found the highest HR for wounds with a lesion score of 1 and the lowest HR for wounds with a lesion score of 3.

### Secondary analyses

No background conditions were maintained in the models. The disbudding operator was maintained in both models. Veterinary students disbudded 22 calves, with a mean difference in healing rate (in mm/week, compared to veterinarians as the reference group) of −0.13 (−0.62; 0.07 CI, mm/week). Nine calves were disbudded by farmers with a mean difference of −0.28 (−2.32; 0.78 CI, mm/week) compared to calves disbudded by veterinarians. Two students performed the disbudding when the calves were disbudded by students, while two farmers disbudded their own calves. Table 3 displays the baseline results of background data.

**Table 3:** Background data.

Hygiene score^a^	Week 0	Week 1	Week 4
Score 1, *n* (%)	97 (38%)	87 (34%)	32 (13%)
Score 2, *n* (%)	100 (39%)	106 (42%)	121 (47%)
Score 3, *n* (%)	39 (15%)	26 (10%)	65 (25%)
Score 4, *n* (%)	19 (7%)	36 (14%)	37 (15%)
Housing type^b^	Solitary	Group	Varied
*N* (%)	15 (6%)	174 (68%)	66 (26%)
Risk of scraping disbudding wounds^c^		Yes	No
*N* (%)		109 (44%)	144 (56%)
Disbudding iron used		Electric^d^	Gas-operated^d^
		184 (72%)	71 (28%)

All numbers and percentages indicate the number and percentage of calves present in this study and subjected to a certain condition.

a Hygiene was scored per pen. Score 1 = clean and dry pen. Score 2 = clean pen, with wet bedding. Score 3 = limited soiling and a wet pen. Score 4 = a lot of diarrhea. *n* = number.

b Housing type was assessed at all farm visits. Solitary and group housing indicates that the calves were in those conditions during the entire study period. Varied indicates the housing type varied over the course of the study period.

c Calves were recorded as having a risk of scraping the disbudding wounds when they had to pass their head to a feed gate or similar structure for feed or drink.

d Electric disbudder from Lister GmbH, Lüdenscheid, Germany & gas-operated Portasol III, Oglesby & Butler Ltd., Carlow, Ireland.

## Discussion

This field trial was designed to compare wound healing after disbudding treatments using a non-antimicrobial wound spray (KF) and an antimicrobial wound spray (CTC). We found that the wound healing rate of KF was numerically lower than CTC, but the difference was small and considered not inferior to CTC. KF-treated wounds had a statistically significant higher risk of a lesion score > 1 than CTC-treated wounds. Although higher lesion scores were associated with a lower healing rate, the difference in the risk of higher LS was not enough to result in an inferior healing rate. Given the large number of calves and the variety of housing conditions present on participating farms, we think that there is a fair chance that our results can be extrapolated to other farms. We are not convinced that our results will also be found in other climate conditions as these may have an impact on the number of flies and other factors that affect wound healing.

Regarding the composition of the two sprays, KF contains the irritative antiseptic chlorocresol, and its application results in the formation of a protective film, with the antiseptic remaining active for a longer period of time (11). Chlorocresol is likely to increase the LS as an immediate effect while the resulting film on the wound may diminish potential negative environmental effects on HR. CTC contains the alcoholic solvent IPA, which may cause irritation but is very volatile; therefore, its effect on the LS is likely less detrimental to wound healing than chlorocresol. The duration of the antimicrobial effect of CTC is limited; in the treatment of skin infections, application is advised every 12 to 24 h. The impact on HR over the course of 4 weeks is limited.

To satisfy the assumptions of the logistic regression model, lesion scores of 2 and 3 were collapsed. This reduced the distinctive properties of this scale. KF had significantly higher lesion scores than CTC, passing the margin of clinical significance of 10%. Scores higher than 1 are considered “delayed healing” by Huebner et al. (9). This is supported by the results of this study, which revealed that higher LS scores were significantly associated with lower HR. For wounds with LS = 3, this appears self-explanatory as purulent exudate is a sign often found in infected wounds. For LS = 2, this distinction is not as clear as scabs are also considered a normal sign in healthy wound healing (4, 8). Wound phase progression is hindered in all infected wounds and in cases of further trauma and mechanical stress to the wound (4). Burn wounds are particularly challenging because of thermal damage to the surrounding tissue. The central region of disbudding wounds is likely permanently damaged due to heat-associated coagulation necrosis (12). The surrounding region with damaged but still viable tissue is referred to as the zone of stasis. This zone is damaged but still perfused, leading to marked inflammatory response, vascular leakage, and edema. If wound healing progresses poorly, this zone tends to progress toward necrosis, resulting in increasing wound size. Infection in this zone can enhance local inflammation and necrosis (12). This is a potential explanation for the negative wound healing rate of wounds classified as LS = 3. For an explanation as to why LS = 2 demonstrates lower wound healing rates than LS = 1, more research into the scoring system is needed.

In previous studies, disbudding wounds have been found to be painful throughout the entire healing duration (13, 14). A faster healing rate is therefore preferred as it reduces the time a calf is exposed to pain. We arbitrarily defined that the HR of KF-treated wounds was inferior when it differed more than 0.5 mm/week from CTC. We used this threshold because a difference of 0.5 mm/week would result in a wound size difference of 2 mm over the 4-week trial period. Any difference smaller than this would be hard to notice without measuring tools. Furthermore, the results from Huebner et al. (9) and Ridgway et al. (8) indicate that the healing rate increases as wound age progresses. We consider that the time until final wound healing is similar for wounds that differ by less than 0.5 mm/week in the first 4 weeks.

The results of this study confirm that wound infections delay the speed of wound healing. We also found that to prevent one calf from developing a wound with LS > 1, a total of five calves would need to be treated with CTC. We consider that the benefit of using CTC spray does not outweigh the cost of using antimicrobials in inducing AMR. As no control group without topical spray was used, there is also a possibility that no treatment is inferior to both CTC and KF treatments. Further research on wound healing sprays with negative controls is necessary to conclude whether treatment is needed at all.

Based on our results, experience with disbudding appears to affect the healing rate as wounds created by veterinarians healed in our study faster than wounds of students and farmers. As this result was based on only two farmers and two students, this finding may not be fit for extrapolation to other situations.

## Conclusion

We found that the healing rate of KF-treated wounds after disbudding is not inferior to CTC, although the risk of a higher LS was worse for KF. This higher risk may be attributable to the irritative characteristics of chlorocresol. In light of prudent antimicrobial use, our study provides evidence that it is possible, under the circumstances, to forego the use of CTC spray after disbudding without a clinically relevant adverse effect on the wound healing rate. Additional research is recommended to provide robust evidence for the efficacy of disinfecting processes with less irritative antiseptics (such as povidone–iodide) followed by applying a protective film as a preferable method after disbudding.

## Data Availability

The raw data supporting the conclusions of this article will be made available by the authors, without undue reservation.

## Supplementary material

SUPPLEMENTARY FIGURE S1 Participant flow throughout the study.
