# Supplementary figures and images for: A clinical field trial on wound healing after disbudding of dairy calves with or without antimicrobial spray
Source: Front Vet Sci. 2025 Apr 7;12:1445605. doi: 10.3389/fvets.2025.1445605 (PMC12010926; doi:10.3389/fvets.2025.1445605)

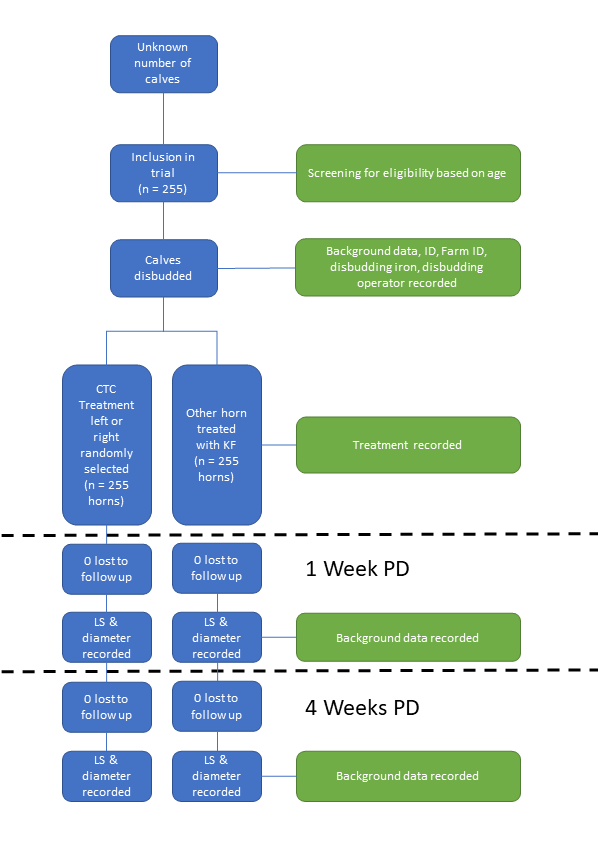

Supplement: SUPPLEMENTARY FIGURE S1 — Participant flow throughout the study. [file Image_1.png]
